# Glial Cells in the Genesis and Regulation of Circadian Rhythms

**DOI:** 10.3389/fphys.2018.00088

**Published:** 2018-02-12

**Authors:** Donají Chi-Castañeda, Arturo Ortega

**Affiliations:** ^1^Laboratorio de Neurotoxicología, Departamento de Toxicología, Centro de Investigación y de Estudios Avanzados del Instituto Politécnico Nacional, Ciudad de Mexico, Mexico; ^2^Soluciones para un México Verde S.A. de C.V., Ciudad de Mexico, Mexico

**Keywords:** circadian rhythms, clock genes, glial oscillators, learning, memory, plasticity

## Abstract

Circadian rhythms are biological oscillations with a period of ~24 h. These rhythms are orchestrated by a circadian timekeeper in the suprachiasmatic nucleus of the hypothalamus, the circadian “*master clock*,” which exactly adjusts clock outputs to solar time via photic synchronization. At the molecular level, circadian rhythms are generated by the interaction of positive and negative feedback loops of transcriptional and translational processes of the so-called “*clock genes*.” A large number of clock genes encode numerous proteins that regulate their own transcription and that of other genes, collectively known as “*clock-controlled genes*.” In addition to the sleep/wake cycle, many cellular processes are regulated by circadian rhythms, including synaptic plasticity in which an exquisite interplay between neurons and glial cells takes place. In particular, there is compelling evidence suggesting that glial cells participate in and regulate synaptic plasticity in a circadian fashion, possibly representing the missing cellular and physiological link between circadian rhythms with learning and cognition processes. Here we review recent studies in support of this hypothesis, focusing on the interplay between glial cells, synaptic plasticity, and circadian rhythmogenesis.

## Introduction

Most light-sensitive organisms have an internal timekeeping mechanism to anticipate daily changes associated with the transition of day to night that is commonly known as “circadian clock”. In 1959, Halberg denominated “circadian rhythms” the biological rhythms that have a period of ~24 h (Halberg, 1959). These rhythms regulate a large number of physiological and behavioral functions in vertebrates, such as hormone secretion, body temperature, metabolism, and even memory processes. The sleep-wake cycle is one the most studied rhythms (Schibler and Sassone-Corsi, 2002; Stratmann and Schibler, 2006; Walker and Stickgold, 2006).

Sleep is a highly conserved process (Hartse, 2011), and several hypotheses support the notion that sleep supersedes learning and memory, possibly through the control of synaptic plasticity (Benington and Frank, 2003; Frank and Benington, 2006; Frank, 2011; Fellin et al., 2012; Frank and Cantera, 2014; De Pittà et al., 2016). Synaptic plasticity refers to the biochemical processes by which synaptic strength changes in an activity-dependent fashion. These cellular cascades are a combination of post-translational modifications that change neural activity, and also result in the reshaping of synaptic terminals (Lohmann and Kessels, 2014). Depending on its temporal course, synaptic plasticity is distinguished into three classes: (1) short-term plasticity, that occurs in the milliseconds to minutes range, and includes the modulation of neurotransmitter release, and depends on post-translational modifications via phosphorylation, ubiquitination, and several other molecular processes (Bliss and Collingridge, 1993; Martin et al., 2000); (2) long-term plasticity, such as long-term potentiation (LTP) and depression (LTD), which may last from hours to months and is represented by cellular changes involving modification of the cellular protein repertoire that may require changes in transcriptional activity and are strictly dependent on protein synthesis (Martin et al., 2000); (3) homeostatic plasticity, which is the result of a variety of molecular and cellular events that shape neuronal circuits, continuously occurs in parallel with other plasticity phenomena and is thought to prevent runaway of neural activity by excessive excitation (Turrigiano, 2011).

Various studies have shown that disruptions of circadian rhythms alter synaptic plasticity and thus, learning and memory, including spatial and place learning and trace fear memory (Winocur and Hasher, 2004; Van der Zee et al., 2008; Wang et al., 2009; Jilg et al., 2010; Kondratova et al., 2010). Based on these studies, it has been suggested that a functional circadian clock is required for optimal learning and memory formation and consolidation (Becker-Weimann et al., 2004; Eckel-Mahan and Storm, 2009). The neural correlates and the mechanisms underpinning these clocks remain largely unknown. In the past two decades, the notion that brain function exclusively relies on neuronal signaling has been challenged by evidence that glial cells work in coordination with neurons, to regulate neurotransmission (Araque et al., 1999). These regulatory events occur through a set of molecular mechanisms that control neurotransmitter recycling (Danbolt et al., 2016), energy requirements (Newman et al., 2011; Suzuki et al., 2011), and eventually sleep homeostasis (Halassa et al., 2009b). This often involves, but is not limited to, the secretion of neuroactive molecules (or “gliotransmitters”) in an activity-dependent manner which target synaptic terminals modulating synaptic transmission (Bergles et al., 1997; Haydon, 2001; Lin and Bergles, 2004; Fellin et al., 2006; Perea et al., 2009).

Interestingly, astrocytes which are the most common glial cells in the cortex, have also been implicated in the circadian control of synaptic plasticity (Lavialle and Servière, 1993; Du et al., 2005; Lavialle et al., 2011; Hayashi et al., 2013a,b), suggesting a possible non-neuronal, glial candidate for the regulation of circadian rhythms that control learning and memory processes. This review focuses on this hypothesis, further elaborating on the possible clinical implications associated with disruptions of glial-mediated pathways on circadian rhythms related to high brain function.

## Circadian clocks and their molecular/genetic bases

Mammalian circadian clocks are hierarchically organized by a “*master clock”* in the suprachiasmatic nucleus (SCN) of the anterior hypothalamus. This clock coordinates independent peripheral clocks (Reppert and Weaver, 2002; Lowrey and Takahashi, 2004). At the molecular level, all of these clocks are the result of a translation-based, interconnected feedback loops in which the transcription factors Brain and Muscle ARNT-Like Protein 1 (BMAL1) and Circadian Locomotor Output Cycles Kaput (CLOCK) form heterodimers that regulate the circadian expression of *Cryptochrome* (*Cry*) and *Period* (*Per;* Dunlap, 1999; Reppert and Weaver, 2001), whose products lead to the inhibition of their own transcription. Additionally, an accessory regulatory loop involves the regulation of *Bmal1* transcription by the coordinated action of the orphan nuclear receptors *Reverse erb* α (*Rev-erb*α, repressor) and *Retinoid-related orphan receptor-*α (*Ror*α, activator) through the binding to the evolutionarily conserved nucleotide sequence [A/T]A[A/T]NT[A/G]GGTCA present in the promoter region of *Bmal1* (Dunlap, 1999; Harmer et al., 2001; Reppert and Weaver, 2001; Preitner et al., 2002).

Significantly, a large number of circadian transcription factors not only regulate their own transcription, but also the expression of numerous other “*clock-controlled genes”* (CCGs) (Dunlap, 1999; Reppert and Weaver, 2001) whose protein products are not essential for the core clock mechanism itself. Among the genes that are part of the CCGs, various enzymes are included, like *phosphoenolpyruvate carboxykinase, glycogen phosphorylase*, and *glucose-6-phosphatase* (Panda et al., 2002); ion channels, like cGMP-gated cation channels, various voltage-gated calcium and potassium channels, the *Na*^+^*/K*^+^*-ATPase*, and a long-opening cation channel (Ko et al., 2009); peptides, such as *arginine-vasopressin* (*Avp*; Jin et al., 1999) and *D element-binding protein* (*DBP*; Le Martelot et al., 2009). In fact, cells rhythmically synthesize about 10% of their transcripts, including those involved in neuronal signaling and synaptic plasticity (Panda et al., 2002; Lowrey and Takahashi, 2004).

## Circadian clocks, sleep, and their involvement in synaptic plasticity

In recent years, numerous reports of *in vitro* and *in vivo* studies, have suggested an essential functional role of sleep in synaptic plasticity (Frank, 2011; Fellin et al., 2012). Accordingly, sleep has been proposed to strengthen, stabilize, or weaken synapses (Benington and Frank, 2003; Frank and Benington, 2006; Frank and Cantera, 2014). The molecular basis of these synaptic changes and whether sleep is necessary for their occurrence remain largely unknown. While sleep is the result of a combination of circadian rhythms and homeostatic mechanisms (Frank and Cantera, 2014), a clear causal connection between circadian clocks, sleep homeostasis, and synaptic plasticity has not been demonstrated.

In this context, it is noteworthy that the recycling of glutamate (Glu) is regulated by clock components, strongly suggesting a functional interplay between circadian rhythms and excitatory synaptic transmission (Beaulé et al., 2009). In fact, glial glutamate transporters are regulated by clock genes having a significant impact in the dynamic, activity-dependent metabolic coupling of glial cells with glutamatergic neurons. This glia/neuron interplay is mediated by the glutamate/glutamine cycle and the astrocyte/neuron/lactate shuttle (Martínez-Lozada and Ortega, 2015). In the same vein, taking into consideration the major role of Glu as the most abundant excitatory transmitter and its role in the molecular models of synaptic plasticity, like LTP and LTD, it is tempting to speculate that another molecular loop between clock genes' expression and glia/neuron coupling via the glutamatergic tripartite synapses control synaptic plasticity at the immediate, mediate and long-term ranges (Flores-Méndez et al., 2016).

## Glial regulation of synaptic plasticity

### Astrocytes

Beyond their recognized role in synapse development and neurodegeneration, astrocytes provide a delicate ensheathment of synapses in the mature brain (Chao et al., 2002; Theodosis et al., 2008). It is well-established that the degree of astrocytic ensheathing greatly changes with the brain area, hinting local specialization. In the hippocampus for example, a single astrocyte is in close proximity to few hundreds dendrites of different neurons, but can ensheathe up to several hundred thousands synapses (Bushong et al., 2002; Halassa et al., 2007; Agulhon et al., 2008). Such morphological arrangement provides the structural substrate for tight functional interactions between astrocytes and neurons (Saab et al., 2012; Bernardinelli et al., 2014).

Astrocytes are also recognized for their role in clearance of neurotransmitters, such as Glu and gamma-aminobutyric acid (GABA), from the synaptic cleft. Perisynaptic astrocytes processes are indeed enriched in transporters that guarantee rapid and efficient removal of the released neurotransmitters (Anderson and Swanson, 2000; Conti et al., 2004). Interestingly, the regulation of the kinetics and the extent of neurotransmitter clearance by astrocytes have been related to synaptic plasticity insofar as they both affect the degree of postsynaptic activation and desensitization (Tzingounis and Wadiche, 2007). Moreover, through the release of a variety of neuroactive molecules, such as Glu, D-serine, adenosine triphosphate (ATP), adenosine, GABA, tumor necrosis factor-α (TNFα), prostaglandins, proteins and peptides, astrocytes are capable of regulating synaptic transmission and plasticity (Halassa and Haydon, 2010; Araque et al., 2014). These neuroactive molecules activate extrasynaptic metabotropic and ionotropic receptors, modifying neurotransmitter release and regulating short-term plasticity and synaptic efficacy (Parpura et al., 1994; Araque et al., 1998a,b, 2014; Halassa et al., 2009a; Halassa and Haydon, 2010).

Increasing evidence indicates that astrocytes could be involved in the synchronization of cortical firing. Cortical circuits for sensory integration are known to display transient synchrony of neuronal ensembles (Harris et al., 2003; Haider and McCormick, 2009). The hallmark of this synchronized activity is the alternation of UP states—i.e., episodes of persistent neuronal firing lasting few milliseconds—and DOWN states—i.e., episodes of neuronal hyperpolarization (Steriade et al., 2001; Brecht and Sakmann, 2002; Cossart et al., 2003; Kenet et al., 2003). UP and DOWN states are common in a wide range of conditions, including quiescent wakefulness (Gentet et al., 2010), anesthesia (Steriade et al., 1993; Ramaswamy and Muller, 2015), and sleep itself (Massimini and Amzica, 2001). Moreover, astrocytes have been implicated in UP state genesis through the release of D-serine, adenosine and ATP (Fellin et al., 2009, 2012; Halassa et al., 2009b; Poskanzer and Yuste, 2011, 2016). Interestingly, gliotransmission has been proposed to operate on different time scales (Fellin et al., 2012). According to the Hill and Tononi's model of sleep and in agreement with the modulation cortical UP and DOWN states, Fellin and colleagues have demonstrated that the depolarizing effect of NMDA receptors currents preserves the UP state (Hill and Tononi, 2004; Fellin et al., 2012). Such a role for NMDA receptors is thought to be dependent on the availability of glia-released D-serine (Fellin et al., 2012), again demonstrating a prominent role of glia/neuron coupling.

### Oligodendrocytes

Oligodendrocytes projections wrap neuronal axons forming the myelin sheaths in the central nervous system (CNS). These myelin sheaths insulate the fibers, and help them to carry the nerve impulses. Interestingly, myelin can influence conduction velocity of the electrical impulse by regulating the axon diameter, thickness of the myelin sheath, the number and spacing of nodes of Ranvier, and nodal structure and molecular composition of ion channels in the node and paranodal region (Berthold et al., 1983; Wurtz and Ellisman, 1986; Baker and Stryker, 1990; Carr and Konishi, 1990; Dupree et al., 2004). Taking this into a consideration, it has been shown that myelin specific proteins, including Nogo-A (Chen et al., 2000; GrandPré et al., 2000), myelin-associated glycoprotein (MAG; McKerracher et al., 1994) and oligodendrocyte-myelin glycoprotein (OMgp; Wang et al., 2002; Huang et al., 2005), inhibit directly axon sprouting and synaptogenesis and constrain nervous system plasticity. This finding indicates the participation of myelin in learning, memory, and cognition.

### Microglia

These glial cells are part of the brain's immune system and are mainly involved in the phagocytosis of foreign matter and cellular wastes of the CNS (Aloisi, 2001). Moreover, during postnatal development and adaptation to novel environments, microglia has a critical role in synaptic remodeling through the elimination of synapses and axon terminals. Additionally, increasing evidence points out that microglia could regulate synaptic plasticity and neurotransmission through the release of gliotransmitters (Batchelor et al., 1999, 2002; Zhong et al., 2010; Harry and Kraft, 2012; Sierra et al., 2013), as well as an increase hippocampal LTP and NMDA receptor-mediated responses via the secretion of glycine (Thomson et al., 1989; Abe et al., 1990; Hayashi et al., 2006). During neuroinflammation, microglia is capable to regulate excitatory neurotransmission by the rapid production of small amounts of ATP, that in turn, recruit astrocytes to augment ATP formation and Glu exocytosis enhancing synaptic transmission via metabotropic Glu receptors (Pascual et al., 2012). In fact, several reports reveal that some of the established astrocytic functions are regulated by the upstream activation of microglia (Ben Achour and Pascual, 2010; Pascual et al., 2012).

## Circadian modulation of the synaptic plasticity in glial cells

Since 1978, it has been demonstrated that diverse cognitive processes are regulated by the circadian clocks in a phase-specific manner (Monk and Folkard, 1978). Particularly, in long-term memories generated in diverse learning paradigms, a role for the endogenous circadian clock has been reported both in vertebrates and invertebrates (Rudy and Pugh, 1998; Valentinuzzi et al., 2004; Rawashdeh et al., 2007). However, the circadian modulation of short-term memory formation has been almost impossible to prove. In this section, we summarize the evidence that involves different glial cells in processes of synaptic plasticity regulated by circadian generators.

### Astrocytes

Gliotransmission is the process by which astrocytes interact with nearby neurons via the release of transmitters, like ATP and Glu (Haydon, 2001; Perea et al., 2009; Parpura and Zorec, 2010). Remarkably, ATP has been linked to modulation of LTP but also of synaptic depression (Gordon et al., 2005; Pascual et al., 2005; Bains and Oliet, 2007). *In vivo*, an astrocyte-dependent rhythmic ATP release is present in the SCN. Although the mechanisms responsible for these ATP oscillations are unknown, calcium-dependent signaling seems to be involved (Womac et al., 2009). Subsequently, it was shown that astrocytes display daily extracellular ATP oscillations that depend on the clock genes (*Clock, Per*, and *Bmal1*) and in inositol triphosphate (IP_3_) signaling, indicating that extracellular ATP levels increase at a specific time of day and suggest a clock-induced increase in energy metabolism and glial activity, which participate in sleep-wake changes in the brain and in control synaptic transmission (Marpegan et al., 2011).

To date, there is no report demonstrating that the circadian clock regulates Glu release. In contrast, accumulating evidence indicates that the glutamate/aspartate transporter (*Glast*) gene expression and protein levels exhibit a diurnal rhythm in a light/dark 12/12 h cycle (Spanagel et al., 2005). These findings are consistent with the absence of rhythmicity of GLAST in the *Per2* mutant mice, pointing out the presence of a circadian control (Spanagel et al., 2005). Later on, using cultured cortical astrocytes from *Clock* mutant animals, it was observed a marked decrease in *Glast* mRNA and protein levels, proposing that glial Glu uptake activity is a function of the clock genes: *Clock, Npas2*, and *Per2* (Beaulé et al., 2009). Specifically, the dependence related to CLOCK and NPAS2 may be due to their involvement in *Glast* transcription, or in GLAST stability and/or localization (Danbolt, 2001). It is important to mention that no conclusive evidence has been shown for a circadian-dependent change in Glu uptake, suggesting a non-circadian role for clock proteins in *Glast* transcription or *Glast* mRNA translation and/or stability (Beaulé et al., 2009). However, it is clear that the regulation and precise function of this transporter is very important to guarantee an efficient glutamatergic neurotransmission. A failure in synaptic Glu clearance is neurotoxic due to a hyperactivation of postsynaptic Glu receptors resulting in the phenomena known as excitotoxicity, which is implicated in most of neurodegenerative diseases (McEntee and Crook, 1993; Domingues et al., 2010; Gegelashvili and Bjerrum, 2014).

In the adult brain, the distribution of the specific astrocyte marker, glial fibrillary acidic protein (GFAP), has been reported to peak during daily rhythms in the SCN (Lavialle and Servière, 1993). Furthermore, it has been demonstrated that this peak also prevails in constant darkness (Lavialle and Servière, 1993; Moriya et al., 2000), strongly suggesting that these rhythms are essential and independent of environmental light. Although the function of circadian fluctuations of GFAP immunoreactivity is unknown, it has been observed that mice lacking the *gfap* gene show reduced eyeblink training and impaired LTD in the cerebellum (Shibuki et al., 1996), suggesting that this protein plays a role in the regulation of neuronal functions.

CNS excitatory synapses are extremely dynamic structures that show stabilization in response to learning and memory process. These synapses are surrounded by intricate astrocytic processes denominated perisynaptic astrocytic processes (PAPs; Iino et al., 2001; Hirrlinger et al., 2004; Nishida and Okabe, 2007). It has been described in primary cultured astrocytes that ezrin (an actin-binding protein) is required for filopodia formation and motility of PAPs, such motility can be induced by Glu via activation of metabotropic Glu receptors 3 and 5 (Lavialle et al., 2011). Moreover, changes in glutamatergic circadian activity in the hamster SCN are in synchrony with changes in ezrin immunoreactivity which is consistent with Glu-induced perisynaptic glial motility (Lavialle et al., 2011). These results suggest that ezrin is essential for Glu-induced PAPs plasticity that could be regulated by circadian system.

On the other hand, the brain fatty-acid binding protein (FABP7) localizes in astrocytes and neuronal cell precursors in the mature brain, and presents a high binding affinity to long chain fatty acids whose effects on brain function include development, emotion, learning, and memory (Yamamoto et al., 1987; Jensen et al., 1996; Wainwright et al., 1997; Moriguchi et al., 2000; Takeuchi et al., 2003). Gerstner and colleagues demonstrated in adult murine brain that levels of *Fabp7* mRNA oscillate over a 24 h period in brain areas that participate in daily activity and sleep like the hypothalamus, the tuberomammilary nucleus, the pons and the *locus coeruleus*. In these areas, *Fabp7* diminish in the dark phase and increase instead in the light phase (Gerstner et al., 2006, 2008). In contrast with its mRNA levels, FABP7 levels are higher during the dark phase (Gerstner et al., 2008), indicating that expression of this protein is delayed by 12 h with regards to its mRNA. More recently, the same group demonstrated that FABP7 is specifically augmented in the perisynaptic compartment of fine astrocytic processes that surround synapses. Furthermore, CPEB-mediated cytoplasmic polyadenylation controls the diurnally regulated *Fabp7* mRNA levels (Gerstner et al., 2012). Accordingly, in plasticity terms, targeting of *Fabp7* and CPEB-mediated polyadenylation could participate in controlling astrocytic process extension, although it is unknown if variations in synaptic plasticity and/or neuronal activity modify polyadenylation and trafficking of *Fabp7* mRNA resulting in morphological modifications of the astrocytic processes (Gerstner et al., 2012). In addition, variations in cycle-dependent memory formation and synaptic plasticity could regulate the circadian variations in subcellular trafficking and localization of *Fabp7* mRNA in hippocampal tripartite synapses (Gerstner et al., 2009, 2012).

### Oligodendrocytes

To date, there is no evidence that oligodendrocytes have an internal circadian clock; however, it has been reported that oligodendrocytes precursor cells (OPCs) proliferation in the hippocampus could be regulated by clock genes (Matsumoto et al., 2011). It should be noted that OPCs proliferation itself could modulate the synaptic plasticity of the hippocampus in response to neuronal activity, thus circadian proliferation of these cells could regulate hippocampal function. Particularly, the OPCs give rise to mature oligodendrocytes, and are thought to be a constitutive reservoir of oligodendrocytes that replace damaged myelin (Levine et al., 1993) or add *de novo* myelination (McCarthy and Leblond, 1988). Interestingly, *myelin proteolipid protein* (*plp*), a myelin-specific gene, is regulated by *Clock* (Du et al., 2005), suggesting that the circadian clock controls myelin formation.

### Microglia

Microglial cells constantly retract and extend their processes to sense their local environment contributing to the maintenance of healthy neuronal circuits (Kirchhoff, 2013). There is evidence that an intrinsic molecular clock exists in cortical microglia which controls diurnal morphological changes of its processes, and whereby these cells regulate the sleep-wake cycle-dependent changes in synaptic strength (Hayashi et al., 2013a,b). In line with these findings, it has been reported that the microglia-specific lysosomal cysteine protease Cathepsin S (CatS) exhibits a circadian expression in cortical microglia. The expression of CatS is involved in diurnal variations of synaptic strength in cortical neurons via the proteolytic modification of the perineuronal environment. However, disruptions in CatS lead to hyperlocomotor activity and to the deletion of the diurnal variations in spine density and synaptic activity of these cortical neurons as a consequence of the failure to downscale synaptic strength during sleep (Hayashi et al., 2013a). Since downscaling of synaptic strength is required for the acquisition and consolidation of novel information upon awakening, it is evident that dysfunction of the microglial intrinsic circadian clock is involved in neuropsychiatric disorders based on sleep disturbance, including depression and cognitive impairment (Bhattacharjee, 2007; Hayashi et al., 2014).

On the other hand, microglial cells express ATP receptors of the P2X (P2XR, ligand-gated ion-channel receptor) and P2Y subtypes (P2YR, G protein-coupled receptor). ATP released by glial cells during neuronal activity is then, capable to influence synaptic transmission. In fact in microglial cells, ATP increases the number their branch points, extension of their processes and morphological complexity (Fontainhas et al., 2011). Specifically, it has been demonstrated that the degree of microglial process extension is controlled by microglial P2Y_12_Rs (Haynes et al., 2006). Moreover, Hayashi and colleagues reported that microglial P2Y_12_Rs present circadian oscillations regardless that microglia would be isolated under constant darkness conditions (Hayashi et al., 2013b). Interestingly, inhibition of these purinergic receptors disrupts the rhythmic patterns of synaptic strength or spine density, while upregulated P2Y_12_Rs during the dark phase results in extension of the microglial processes that are partially retracted during the light phase resulting in a decrease of synaptic strength or spine density (Hayashi et al., 2013b). In the same fashion of CatS disruptions, dysfunctions in microglia-synapse interactions participate in neuropsychiatric disorders (Bhattacharjee, 2007; Hayashi et al., 2014).

Concerning P2X purinergic receptors, Nakazato and coworkers demonstrated that ATP selectively promotes the expression of the protein and mRNA of *Per1* through the activation of P2X7R in microglial cells (Nakazato et al., 2011). While the outcome of this upregulation is not completely clear, it has been reported that *Per* is not only crucial for long-term memory formation (LTM), but overexpression of this gene also enhances memory formation (Sakai et al., 2004). Taken together these evidences suggest that *Per* has an important function in the regulation of circadian synaptic plasticity in microglia.

Additionally, several reports indicate that ATP promotes microglial cells to secrete several signaling molecules, like interleukin-1 beta (IL-1β), TNFα, and plasminogen (Inoue et al., 1998; Hide et al., 2000; Sanz and Di Virgilio, 2000; Suzuki et al., 2004); which are involved in the modulation of synaptic transmission and plasticity (Ikegaya et al., 2003; Becker et al., 2013; Liu et al., 2014). Finally, it should be noted that microglia display rhythmic fluctuations in the gene expression of these mediators (Fonken et al., 2015).

## Clinical implications

Although synaptic dysfunction is the cellular basis of most mental illnesses, disturbance of the circadian clock system and dysfunctions of glial cells are likely to be involved in diverse brain pathologies. Up to now, only few studies, summarized in Table 1, provide a link between brain disorders, circadian rhythm dysfunction and glial physiology. For example, a study using *Per2*^*Brdm*1^ mutant mice demonstrated that a nonfunctional *Per2* results in a hyperglutamatergic state due to a reduced GLAST expression and as a consequence, Glu uptake by astrocyte is diminished (Spanagel et al., 2005). Accordingly, one could expect that a reduction of astrocytic Glu uptake would be related to severe pathophysiological implications as shown in several disease models, including multiple sclerosis, Alzheimer's, and Huntington diseases (Domingues et al., 2010). Additionally, several studies related to proteins that are involved in the regulation of the astrocytic processes extension that surround synapses, like ezrin and FABP7, have shown that dysfunctions in these proteins lead to impairment in processes, like development, learning, memory, and emotion (Lavialle et al., 2011; Gerstner et al., 2012).

**Table 1:** Clock-controlled genes (CCG) and their implications in brain pathologies.

**CCG**	**Preparation**	**Pathological implications**	**References**
**ASTROCYTES**			
ATP	Cortical astrocyte cultures	Disruptions in sleep-wake changes in the brain and in control synaptic transmission.	Marpegan et al., 2011
GLAST	*Per2* mutant mice	Dysregulation in the Glu uptake process.	Spanagel et al., 2005
GFAP	GFAP mutant mice	Impaired LTD in the cerebellum, as well as reduced eyeblink conditioning.	Shibuki et al., 1996
Ezrin	Primary astrocytes cultures	Alterations in the Glu-induced PAPs plasticity.	Lavialle et al., 2011
FABP7	Primary mouse astrocyte culture	Dysregulation of astrocytic processes extension.	Gerstner et al., 2012
**OLIGODENDROCYTES**			
OPCs	Mouse hippocampus slices	Alterations in synaptic plasticity for the hippocampal function.	Matsumoto et al., 2011
**MICROGLIA**			
CatS	CatS^−/−^ mice	Neurological disorders by disruption of the circadian oscillation patterns of synaptic strength and spine density in cortical neurons.	Hayashi et al., 2013a
P2Y_12_R	Cortical microglia cultures	Neurological disorders by disruption of the rhythmic patterns of synaptic strength or spine density.	Hayashi et al., 2013b
P2X7R	Cultured murine microglia and BV-2 cells	Downregulates *Per1* mRNA expression.	Nakazato et al., 2011
		Reduces the number of processes in microglial cells as a result of cellular activation.	

*ATP, Adenosine triphosphate; CatS, cathepsin S; FABP7, brain-type fatty acid binding protein; GFAP, glial fibrillary acidic protein; GLAST, Glu/aspartate transporter; LTD, long-term depression; OPCs, oligodendrocytes precursor cells; P2X7R, P2X7 receptor; P2Y_12_R, P2Y_12_ receptor; PAPs, perisynaptic astrocytic processes*.

The fact that microglia exhibits circadian rhythmicity, such as oscillating expression patterns of clock genes that regulate the expression of P2Y_12_R and of the CatS protease suggests that alteration of these two factors disrupts the rhythmic patterns of synaptic strength and spine density (Hayashi et al., 2013a,b). In this context, healthy brain synaptic homeostasis depends on microglia-synapse interactions controlled by the intrinsic microglial clock, so the dysfunction of this clock most probably leads to neuropsychiatric disorders, like depression and cognitive deficits (Bhattacharjee, 2007; Hayashi et al., 2014).

Finally, it has been demonstrated that sleep disturbances are involved with multiple negative effects on human physiology, including neuronal dysfunction (Joo et al., 2013), mood disturbances (Dinges et al., 1997), cognitive impairments (Lo et al., 2012), and disruption to circadian rhythmicity (Möller-Levet et al., 2013).

## Conclusions

Glial cells have long been regarded as simple supportive cells of neuronal function. However, in recent years, several reports have demonstrated the involvement glial cells in diverse processes required for proper brain function, including contribution to the regulation of the synaptic plasticity. Taking into consideration that clock genes modify glial Glu transporters and, by these means, control the strength and continuity of the major excitatory system, their role in higher brain functions is likely of a high relevance. Accordingly, specific alterations of the circadian system are related to various diseases in which glutamatergic transmission is impaired. Additionally, dysfunction of astrocyte-neuron signaling plays a critical role in the pathology of most of the neurodegenerative diseases, such as Alzheimer, Parkinson, and Huntington. Altogether, these findings make it clear that glial cells are an important tool to understand the circadian regulation of synaptic plasticity, both in the short and in the long terms. Certainly, characterization of the activity-dependent and clock-dependent changes in glial proteins repertoire will provide a major input to our understanding of the pivotal role of glial cells in higher brain functions.

## Author contributions

DC-C gathered the relevant information, wrote the manuscript, as well as elaborated the table. AO reviewed and edited the final version of the manuscript.

### Conflict of interest statement

The authors declare that the research was conducted in the absence of any commercial or financial relationships that could be construed as a potential conflict of interest.
